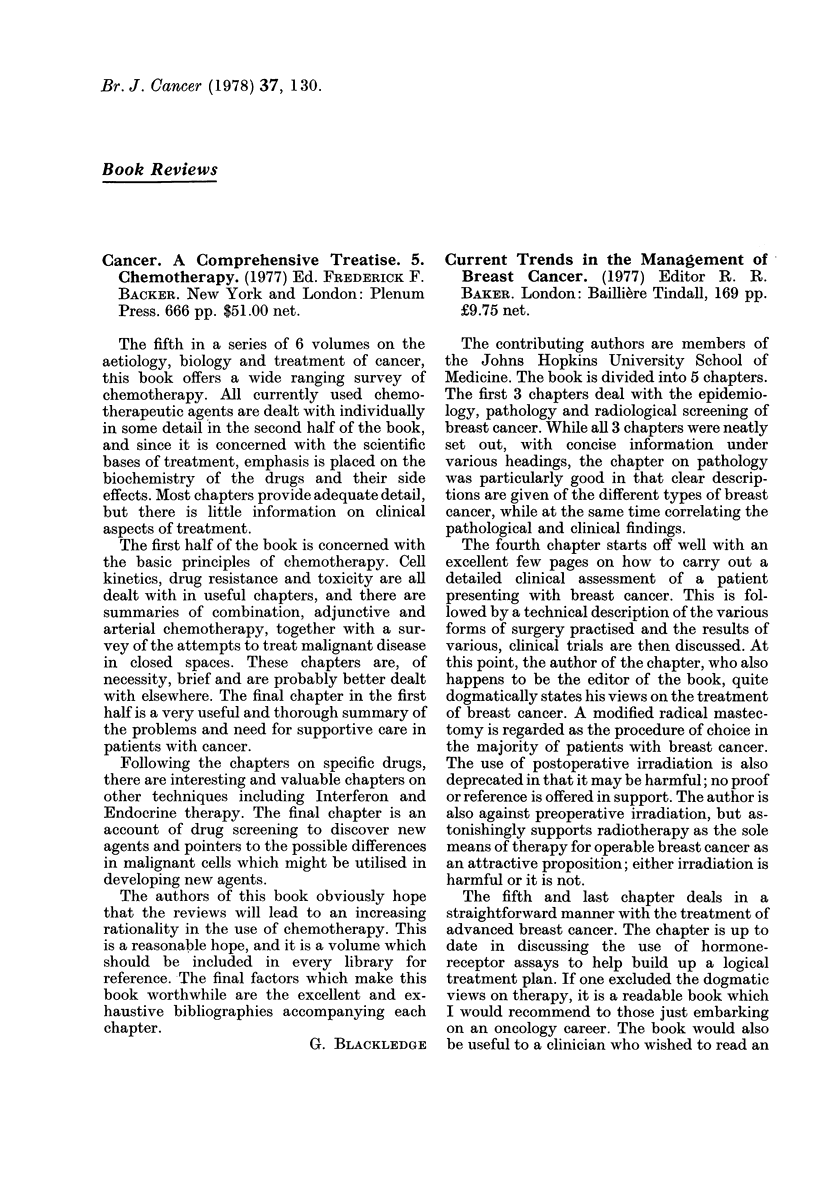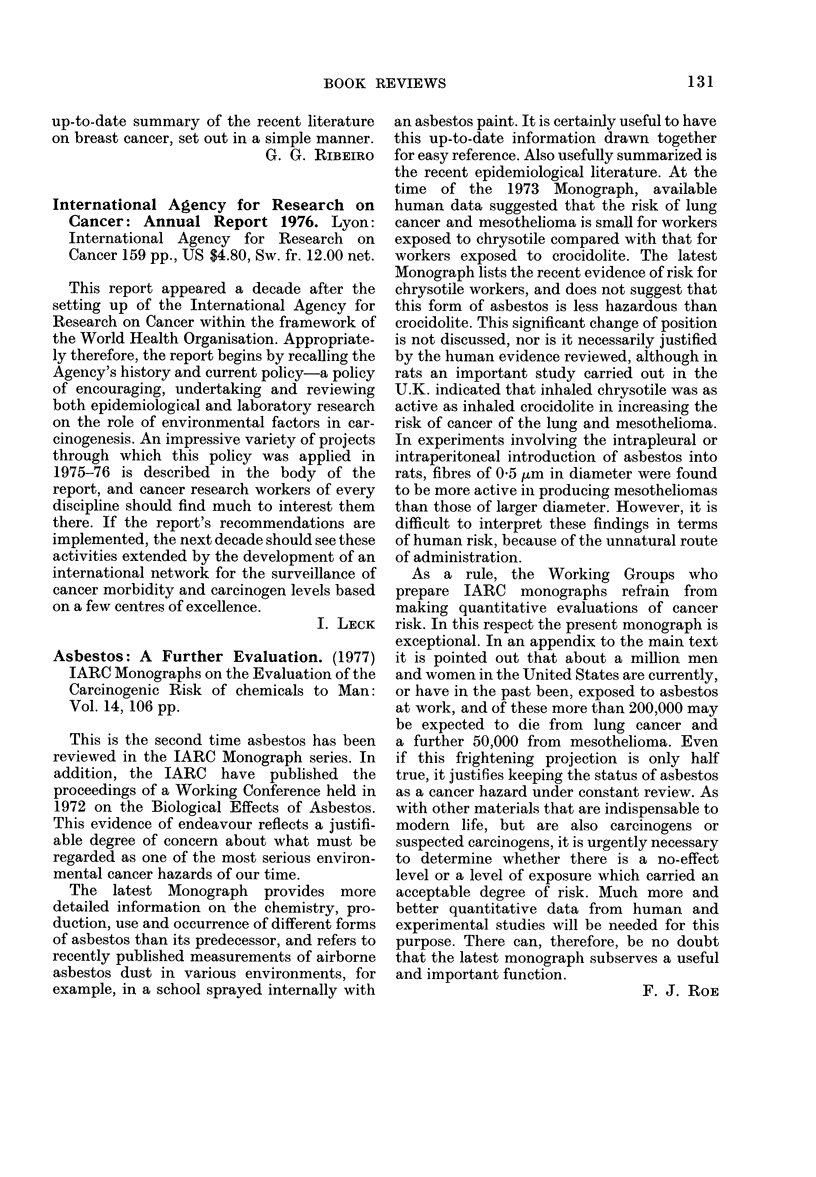# Current Trends in the Management of Breast Cancer

**Published:** 1978-01

**Authors:** G. G. Ribeiro


**Current Trends in the Management of Breast Cancer.** (1977) Editor R. R. BAKER. London: Baillière Tindall, 169 pp. £9.75 net.

The contributing authors are members of the Johns Hopkins University School of Medicine. The book is divided into 5 chapters. The first 3 chapters deal with the epidemiology, pathology and radiological screening of breast cancer. While all 3 chapters were neatly set out, with concise information under various headings, the chapter on pathology was particularly good in that clear descriptions are given of the different types of breast cancer, while at the same time correlating the pathological and clinical findings.

The fourth chapter starts off well with an excellent few pages on how to carry out a detailed clinical assessment of a patient presenting with breast cancer. This is followed by a technical description of the various forms of surgery practised and the results of various, clinical trials are then discussed. At this point, the author of the chapter, who also happens to be the editor of the book, quite dogmatically states his views on the treatment of breast cancer. A modified radical mastectomy is regarded as the procedure of choice in the majority of patients with breast cancer. The use of postoperative irradiation is also deprecated in that it may be harmful; no proof or reference is offered in support. The author is also against preoperative irradiation, but astonishingly supports radiotherapy as the sole means of therapy for operable breast cancer as an attractive proposition; either irradiation is harmful or it is not.

The fifth and last chapter deals in a straightforward manner with the treatment of advanced breast cancer. The chapter is up to date in discussing the use of hormone-receptor assays to help build up a logical treatment plan. If one excluded the dogmatic views on therapy, it is a readable book which I would recommend to those just embarking on an oncology career. The book would also be useful to a clinician who wished to read an up-to-date summary of the recent literature on breast cancer, set out in a simple manner.

G. G. Ribeiro